# Preface

**DOI:** 10.1155/EDR.2003.201

**Published:** 2003


*Experimental Diab. Res.*, 4:201, 2003
Copyright © Taylor and Francis Inc.
ISSN: 1543-8600 print / 1543-8619 online
DOI: 10.1080/15438600390249655

### Preface

The material of this special issue of *Experimental Diabesity Research* is focused on the increasing awareness of perturbations of growth factors, vasoactive substances, and interleukins in the pathogenesis of the microvascular complications of diabetes. The elucidation of these abnormalities and their relationship to the complex dynamic pathogenetic components underlying the devastating consequences of diabetic complications will provide inroads to the development of preventive and therapeutic measures. In the introductory paper, Derek Le Roith gives a comprehensive overview of the biological function of the various components of the insulin-like growth factor (IGF) system and how these components are controlled under physiological and pathophysiological conditions. Liam J. Murphy reviews the role of IGFs, and IGF binding proteins in glucose homeostasis and their interactions with insulin action. An up-to-date overview of the role of various growth factors and cytokines in the progressive development of diabetic kidney pathology is expertly provided by Frank C. Brosius III. The role of IGFs, insulin, and the recently elucidated biological effects of the proinsulin C-peptide and their interactions with respect to various mechanistic components of diabetic polyneuropathy and primary encephalopathy is reviewed by Anders A. F. Sima and colleagues. Douglas Ishii and Sean Lupien provide a comprehensive and expert compilation of the potential therapeutic usefulness of IGFs in the treatment of peripheral and central nervous complications of diabetes. The perturbations of nerve growth factor (NGF) and related neuropeptides in diabetic neuropathy are updated by Gary Pittinger and Aaron Vinik, who also critically review the potential therapeutic value of this neurotrophic factor. Drs. Kahn and Chakrabarti deal with proliferative diabetic retinopathy and the pathogenetic significance of growth factors and angiogenic factors in a comprehensive overview. The increasingly recognized importance of cytokines and interleukins and their relationships to the pathogenesis of diabetic neuropathy is brought up-to-date by Dusanka S. Skundric and Robert P. Lisak.

This special issue is intended to provide a review of the important participation of abnormalities of growth factor, including insulin and the proinsulin C-peptide themselves, and cytokines in the microvascular complications of diabetes. This evolving area of research into the complications is increasingly being acknowledged and is likely to provide new avenues for the development of new and renewed preventive and therapeutic pharmaceutical approaches to the debilitating complications of diabetes.

I wish to thank the authors for the fine contributions and hope that this special issue will be a useful publication for anybody involved in the combat of diabetes and its complications.

*Anders A. F. Sima*
*Detroit, August 2003*

